# Additional Value of Mini-Cog© in Urogeriatric Patients Concurrently Screened by G8 Scores

**DOI:** 10.3390/medicines10100056

**Published:** 2023-10-08

**Authors:** Jobar Bouzan, Peter Willschrei, Marcus Horstmann

**Affiliations:** 1 Department of Urology, University of Duisburg-Essen, Hufeland Str. 55, 45147 Essen, Germany; jobar.bouzan@krankenhaus-dueren.de; 2 Department of Urology, Helios Hospital St. Josefshospital, Krefeld Uerdingen, Kurfuersten Str. 69, 47829 Krefeld, Germany; 3 Department of Geriatrics, Evang. Hospital Essen-Steele, Am Deimelsberg 34, 45276 Essen, Germany; 4 Department of Urology, Klinikum Guetersloh, Reckenberger Str. 19, 33332 Gütersloh, Germany

**Keywords:** geriatric screening, geriatric assessment, G8 score, geriatric urology, Mini-Cog**©**

## Abstract

**Background:** Cognitive impairment is poorly addressed in G8 screening. The aim of the present study was to evaluate the additional value of Mini-Cog© in urogeriatric patients concurrently screened by G8 scores. **Methods:** Seventy-four consecutive urogeriatric patients aged 75 and above were evaluated. All patients underwent G8 and Mini-Cog**©** screening. Patients with a G8 score above 14 were considered geriatric “healthy or fit”. A Mini-Cog© from four to five points was considered inconspicuous in screening for cognitive impairment. The additional information of a Mini-Cog© screening during G8 screening was evaluated by looking at G8 “fit and healthy” patients who had conspicuous Mini-Cog© tests and vice versa. Additionally, the results of the neuropsychological subitem “E” of the G8 score were compared with the results of the Mini-Cog© screening. **Results:** The mean age of the patients was 83 y (min. 75–max. 102). Sixty-one of the patients were males, and 13 were females. Twenty-nine of the patients had a normal G8 score and were considered “healthy or fit”, and 45 were not. Forty-three of the patients had an inconspicuous Mini-Cog©, and 31 had a conspicuous Mini-Cog© of less than four points. The majority of G8 “healthy or fit” patients (*n* = 24/29) had an inconspicuous Mini-Cog© test. However, of them, five patients had a Mini-Cog© of less than four points, which is suspicious for cognitive disorders. Furthermore, of the 43 patients with a normal G8 subscore in item “E” of two points, 6 patients had a conspicuous Mini-Cog© of less than four points. **Conclusions:** As shown by the present study, the Mini-Cog© might extend the G8 screening with regard to the detection of cognitive functional impairments that are not detected by the G8 screening alone. It can be easily added to G8 screening.

## 1. Background

Geriatric aspects are important in urologic patients for further medical decision making and should be assessed [1]. Decisions include whether to perform major surgery for urological malignancies such as prostate, bladder or renal cancer [2] or whether to start chemotherapy for advanced malignancies. Currently, geriatric aspects are best evaluated by a full comprehensive geriatric assessment (CGA) [3,4]. This is time- and resource-consuming and cannot be performed in every patient. As a solution, short screening scores—such as the G8 score—have been developed [5,6].

The G8 score is a questionnaire of eight items [6]. It mainly addresses nutritional aspects that are covered in three items. Other items cover the area of mobility, neuropsychological restrictions, polypharmacy, age and comparison of the subjective health status with peers. Patients who reach more than 14 points of the maximum of 17 points are considered geriatric “fit or healthy”. Patients with 14 or fewer points have a conspicuous test and are suspicious of “frailty”. Even though the G8 score has proven in the past to be a valuable tool in geriatric oncological patients [5,7,8], it has some drawbacks if it is used alone. In addition to its low specificity [9], the cognitive impairments of patients are poorly addressed by the test. Of its eight items, only item “E” addresses neuropsychological aspects. Furthermore, item “E” only relies on the subjective evaluation of the patients themselves and/or the examiner. Even though the G8 score is mostly used in oncologic patients, it was recently likewise evaluated in 200 non-cancer patients by Cavasoglu et al. In their study, it proved to be a valid and reliable tool in patients with benign diseases [10].

In the present study, we added a Mini-Cog© to the G8 screening and evaluated whether this combination gives additional information regarding cognition compared to G8 screening alone. The Mini-Cog© is a screening test for cognition, in which the patient is asked to memorize three words, draw a clock and set the hands to 10 past 11 [11]. A maximum of five points can be reached. If three points are reached, the test is suspicious of mild cognitive impairment, and if two or fewer are reached, the test is suspicious of severe cognitive impairment. The Mini-Cog© is a validated screening tool that has been shown to have a high sensitivity and specificity for identifying cognitive impairment [12].

Currently, the combination of the G8 score and the Mini-Cog© is recommended for geriatric prostate cancer and muscle-invasive bladder cancer patients by the International Society of Geriatric Oncology (SIOG), the European Society of Urology (EAU) and the European Society for Radiotherapy and Oncology (ESTRO) [13,14,15]. Until now, however, the evidence for the combination of both tests in urogeriatric screening remains low. To the knowledge of the authors, there are no published data of patients available in the literature evaluating the combination of both tests in urogeriatric patients. Our Medline research only revealed a few studies in non-urologic patients in which both tests were applied [16,17,18]. Among them is a recent Irish pilot study in which both tests were performed in metastatic cancer patients, but the additional value for the Mini-Cog© was not evaluated [18]. Of course, other screening tests for geriatric frailty and cognitive impairment also might have been evaluated; however, we decided to evaluate the combination of the G8 score and Mini-Cog©, because of its guideline recommendation in urooncology.

## 2. Methods

Seventy-four consecutive patients aged 75 years or older who were treated for urological diseases were included in the study. All patients routinely underwent both a G8 and a Mini-Cog© screening simultaneously at admission to the hospital and were treated at the Department of Urology of the St. Josefshospital in Krefeld Uerdingen between 2019 and 2020. Tests were performed by residents of urology (J.B., B.S., S.N.). A G8 score ≤ 14 points was considered positive for the suspicion of geriatric “frailty”. Patients with a G8 score > 14 were classified as geriatric “fit or healthy”. A Mini-Cog© score of less than 4 pts. was considered suspicious for cognitive impairment. Patient characteristics were gathered from patients’ charts. They included age, sex, reason for admission, comorbidities, length of stay and the number of coded diagnoses. To evaluate if the Mini-Cog© is of additional value during G8 screening, we first looked at the number of patients who were considered G8 “fit and healthy” but had a suspicious Mini-Cog© and vice versa. Then, we compared the test results of the single subjective neuropsychological item of the G8 score (item “E”) to the results of the Mini-Cog© screening. This neuropsychological item “E” of the G8 score consists of three options with a score ranging from zero to two points. Zero points are given if the patient suffers from severe depression or dementia, one point for mild dementia and two points for no problems (see G8 score, Item E) [5]. Descriptive statistics were performed by Excel, Microsoft, 2016, Version 15.34. Comparisons were made by t-tests and Wilcoxon and Kurskall Wallace tests using SPSS software, IBM, Version 27. The level of significance was defined at a p-level of <0.05. The study was performed according to the ethical standards of the Medical Council of North Rhine Westphalia, Germany. It required no ethical approval or informed consent from the patients because of an evaluation of routine data (248/2020).

## 3. Results

The mean age of the patients was 83 years (min. 75 years–max. 102 years). Sixty-one of the patients were males, and thirteen were females. Forty-three of the patients were treated for uro-oncologic malignancies and thirty-one for benign conditions. Patient characteristics are listed in Table 1.

Both tests were easy and quick to perform. Each test took approximately 5–7 min. In the G8 screening, 29 of the patients had inconspicuous and 45 conspicuous results. The mean G8 score of all patients was 12.6 points (range 4–17 pts.). Male patients had a significantly higher G8 score than female patients (13.0 pts. vs. 10.46 pts., *p* = 0.01), and patients with oncological diseases had a higher but not significantly higher G8 score than patients with non-oncologic diseases (13.1 pts. vs. 11.9 pts, *p* = 0.07). The mean G8 score results decreased significantly by age group (*p* < 0.01). Seventy-five- to seventy-nine-year-old patients (*n* = 18) had a mean G8 score of 14.7 pts, 80- to 84-year-old patients had a mean G8 score of 13.4 pts (*n* = 32) and patients above 84 years had a mean G8 score of 10.2 pts (*n* = 25).

Forty-three of all the patients had a normal Mini-Cog© score of four or more, and thirty-one had scores of less than four points. The Mini-Cog© score results were not significantly different between male and female patients (3.4 vs. 3.5 pts, *p* = 0.86). Patients with oncological diseases had a higher but not significantly higher Mini-Cog© score than patients with non-oncologic diseases (3.7 pts. vs. 3.0 pts, *p* = 0.09). The mean Mini-Cog© score results were both 3.6 points in the age groups of 75- to 79-year-old patients (*n* = 18) and of 80- to 84-year-old patients (*n* = 32). Patients above 84 years had a mean Mini-Cog© score of 2.9 pts. This was not significantly different from the other age groups (*p* = 0.18).

The majority of G8 “fit and healthy” patients (*n* = 24/29) had a normal Mini-Cog©. However, five out of twenty-nine G8 “fit and healthy” patients had a Mini-Cog© of less than four points. All five patients were male. Of these patients, two had benign bladder neck stenosis (age 75 years and 85 years), two had symptomatic prostate enlargements (age 79 and 89 years) and one had non–muscle-invasive bladder cancer (age 75 years). Three of them had a Mini-Cog© of three and two of two points. Further clinical evaluation without systematic testing confirmed all significant cognitive disorders. This led to the individual decision that both patients with prostate enlargement and the 85-year-old patient with bladder neck stenosis did not undergo surgical treatment. Of the 45 G8 “frail” patients, 19 had an inconspicuous Mini-Cog© and 26 had a conspicuous Mini-Cog© (Table 2). The mean G8 score of patients with an inconspicuous Mini-Cog^©^ was 13.9 points and of those with a score under four 10.7 points, respectively. Differences between both groups were significant (*p* < 0.05).

Forty-three of all 74 patients reached two points on the neuropsychological item “E” and had no suspicion for neuropsychological impairment according to the screening test (Table 3). Thirty-one lost two points or one point in this item and had a conspicuous sub-item “E” test result. Eleven of the thirty-one patients lost two points and twenty lost one point. In comparison to the Mini-Cog© screening, 6 out of 43 patients with a normal score in sub-item “E” had a conspicuous Mini-Cog© of below four points. Three of them were in the abovementioned group of patients with an inconspicuous G8 score. They were both patients with prostate enlargement, and one patient had bladder neck stenosis. Of the three other patients, two patients had metastatic prostate cancer (age 82 years and 83 years), and one at the age of 84 years also suffered from benign prostate enlargement.

## 4. Discussion

In the present study, we evaluated G8 screening in combination with Mini-Cog© screening in our clinical routine, as it is currently recommended by the International Society of Geriatric Oncology (SIOG), the European Society of Urology (EAU) and the European Society for Radiotherapy and Oncology (ESTRO) [13,14,15] for geriatric prostate cancer and muscle-invasive bladder cancer patients. In our study, we applied both tests at hospital admission as a geriatric screening in a heterogeneous group of patients, including patients with non-oncologic conditions, and we evaluated whether there was additional information regarding the domain of cognition by adding a Mini-Cog© screening. First, we recognized that both screening tests were easy and quick to perform in most patients within a time period of 5–7 min per test. Next, similar to other screening cohorts, we found a high number of G8 “frail” patients in our study groups. This is in line with the literature, in which the G8 score is known to be a very sensitive test at the price of a low specificity [8,9,19,20].

Whereas the G8 score is a general geriatric screening score for geriatric risk factors mainly designed for oncologic patients, the Mini-Cog© screens for cognitive impairment alone. It is an objective short assessment in which the cognitive performance of the patient is measured by evaluating the memory of three words and drawing a clock and setting the hands to 10 past 11. This is in contrast to the single sub-item “E” of the G8 score, which also screens for neuropsychiatric disorders but in a subjective manner.

Even though both the G8 score and the Mini-Cog© aim to screen different geriatric aspects, the main question in the evaluation of our patients was how big the congruence between G8 and Mini-Cog© screening was and if the use of the G8 score alone would be sufficient to also screen for cognitive impairment. In general, we found that the majority (83%) of patients who were G8 “fit and healthy” also had a normal Mini-Cog©. However, five patients (17%) had a normal G8 score but a conspicuous Mini-Cog©. All of them were males and candidates for transurethral resection of either the bladder or the prostate. Further clinical evaluation confirmed significant cognitive disorders in all of them, with the consequence that in an individual treatment approach transurethral resections were not performed in three of them. However, in other individual patients even with cognitive impairment, transurethral resection might have been an option in order to avoid long-term catheterization. In our eyes, this is a relevant number of patients and proves that an additional cognitive screening is helpful for further decision making in patients who undergo a G8 screening.

Furthermore, the majority of patients who had an inconspicuous subscore on item “E” of the G8 score also had an inconspicuous Mini-Cog© (*n* = 37/43, 86%). This high congruence was surprising to us because the G8 screening is only based on anamnestic findings and/or self-information of the patients, whereas the Mini-Cog© objectively measures cognition. Of the patients with a normal subscore, however, six (14%) still had a conspicuous Mini-Cog©. Three of these patients were also in the abovementioned group of five patients with a normal G8 score but a conspicuous Mini-Cog©. These results further demonstrate that additional Mini-Cog© testing is of value for a general patient assessment in this setting because cognitive deficits might otherwise have been missed.

Basically, geriatric screening was used in our group of patients to better assess patients. At the time of the study geriatric screening was not included in our treatment algorithms, because it was not yet part of a fix clinical routine at that time. However, already at that time, it served as an active treatment confirmation, like, for example, surgery in geriatric “fit” patients and treatment modifications, as described above, in some “unfit” patients. In the future, the main goal of a geriatric assessment, in our eyes, should be to reduce the complications and risks of treatment by identifying patients who are at risk and need further evaluation.

The major limitation of the present study is that both screening tests were not systematically evaluated by a simplified geriatric assessment or a full comprehensive geriatric (CGA) for general geriatric aspects and a Mini-Mental-Status-Test or Montreal-Cognitive-Assessment for cognition but only by clinical evaluation. Another major limitation is that our group of patients was relatively small, heterogenous and did not exclude non-oncologic patients. These limitations do not allow us to draw major conclusions from our study. However, the heterogeneity of our group of patients reflects everyday clinical practices in urology, in which we realized, that geriatric screening is not only important for oncologic but also for non-oncologic decision making. Even though the G8 score has been until now mainly used in oncologic patients, Cavasoglu et al. recently showed in 200 non-cancer patients that it is also a valid and reliable tool [10]. In this context, we consider our evaluation of interest and value.

## 5. Conclusions

The present study revealed that important deficits of cognition would have remained undiscovered if only a G8 score had been performed. This supports the concept that Mini-Cog© screening might give additional information to G8 screening and be of clinical relevance in clinical decision making. Our results support the current guidelines that recommend an additional Mini-Cog© during G8 screening [13,14,15]. In our eyes, when cognition is being addressed, it is not only important to choose the optimal treatment for the patient but also to assess the patient’s capacity to evaluate information and make informed decisions [14].

## Figures and Tables

**Table 1:** Patients’ characteristics, NMIBC: non–muscle-invasive bladder cancer, MIBC: muscle-invasive bladder cancer, * afebrile patients without systemic inflammation.

	All
patients (m/f), (*n* = pat.)	74 (61/13)
age (mean y, min–max)	83 (75–94)
prostate cancer	24
*localized*	*14*
*metastatic*	*10*
urothelial cancer	16
*NMIBC*	*10*
*MIBC, metastatic, upper tract*	*6*
renal cancer	4
*localized*	*4*
urolithiasis	4
urinary tract infection *	8
benign prostate enlargement	15
ureteral stenosis	3
G8 score (mean (min-max))	12.6 (4–17)
Mini-Cog© (mean (min-max))	3.3 (0–5)
Charlson comorbidity score (mean (min-max))	3.3 (0–7)

**Table 2:** Results of Mini-Cog© screening compared to results of G8 screening.

	Mini-Cog^©^ (Test Results ≥ 4 pts.) “Fit”	Mini-Cog^©^ (Test Results < 4 pts.) “Unfit”
Patients (*n* = 74), %	43 (58%)	31 (42%)
Mean G8 score (pts.)	13.9	10.7
G8 “fit and healthy” patients (*n* = patients)	24	**5**
G8 “frail” patients (*n* = patients)	19	26

**Table 3:** Results of the neuropsychological subscore item “E” of the G8 score compared to Mini-Cog© results.

	Subscore Item “E” (Score 0 Points)	Subscore Item “E” (Score 1 Point)	Subscore Item “E” (Score 2 Points)
Patients (*n* = patients, %)	11 (15%)	20 (27%)	43 (58%)
Mean Mini-Cog© (pts.)	1.63	1.75	4.6
Pat. with a Mini-Cog© ≥4 pts. “fit” (*n* = patients)	3	3	37
Pat. with a Mini-Cog© <4 pts. “unfit” (*n* = patients)	8	17	**6**

## Data Availability

All data generated or analyzed during this study are included in this article. Further inquiries can be directed to the corresponding author.

## Abbreviations

CGA	comprehensive geriatric assessment
SIOG	International Society of Geriatric Oncology
EAU	European Society of Urology
ESTRO	European Society for Radiotherapy and Oncology
